# Quantitative meta-analysis of fMRI and PET studies reveals consistent activation in fronto-striatal-parietal regions and cerebellum during antisaccades and prosaccades

**DOI:** 10.3389/fpsyg.2013.00749

**Published:** 2013-10-16

**Authors:** Sharna D. Jamadar, Joanne Fielding, Gary F. Egan

**Affiliations:** ^1^Monash Biomedical Imaging, Monash UniversityMelbourne, VIC, Australia; ^2^School of Psychology and Psychiatry, Monash UniversityMelbourne, VIC, Australia

**Keywords:** antisaccade, oculomotor, functional magnetic resonance imaging, positron emission tomography, meta-analysis, activation likelihood estimation

## Abstract

The antisaccade task is a classic task of oculomotor control that requires participants to inhibit a saccade to a target and instead make a voluntary saccade to the mirror opposite location. By comparison, the prosaccade task requires participants to make a visually-guided saccade to the target. These tasks have been studied extensively using behavioral oculomotor, electrophysiological, and neuroimaging in both non-human primates and humans. In humans, the antisaccade task is under active investigation as a potential endophenotype or biomarker for multiple psychiatric and neurological disorders. A large and growing body of literature has used functional magnetic resonance imaging (fMRI) and positron emission tomography (PET) to study the neural correlates of the antisaccade and prosaccade tasks. We present a quantitative meta-analysis of all published voxel-wise fMRI and PET studies (18) of the antisaccade task and show that consistent activation for antisaccades and prosaccades is obtained in a fronto-subcortical-parietal network encompassing frontal and supplementary eye fields (SEFs), thalamus, striatum, and intraparietal cortex. This network is strongly linked to oculomotor control and was activated to a greater extent for antisaccade than prosaccade trials. Antisaccade but not prosaccade trials additionally activated dorsolateral and ventrolateral prefrontal cortices. We also found that a number of additional regions not classically linked to oculomotor control were activated to a greater extent for antisaccade vs. prosaccade trials; these regions are often reported in antisaccade studies but rarely commented upon. While the number of studies eligible to be included in this meta-analysis was small, the results of this systematic review reveal that antisaccade and prosaccade trials consistently activate a distributed network of regions both within and outside the classic definition of the oculomotor network.

## Introduction

The antisaccade task (Hallet, 1978) is a classic paradigm in the study of cognitive control that requires participants to inhibit a reflexive saccade and instead make a volitional saccade toward the mirror opposite location. *Antisaccade* trials are often compared to *prosaccade* trials where participants make a visually-guided saccade toward a peripheral target. This task has been studied extensively in the behavioral, electrophysiological, and neuroimaging literature in both non-human primates and humans, as such the basic neural circuitry underlying this task is quite well understood (see Pierrot-Deseilligny et al., 2004; Hutton, 2008; McDowell et al., 2008 for reviews). It has also been studied extensively in psychiatric and neurologically impaired populations such as schizophrenia (e.g., Raemaekers et al., 2002), autism (e.g., Manoach et al., 1997), attention-deficit hyperactivity disorder (ADHD; Mostofsky et al., 2001), Alzheimer's disease (e.g., Kaufman et al., 2010), Parkinson's disease (e.g., Cameron et al., 2012), Huntington's disease (e.g., Blekher et al., 2004), and multiple sclerosis (e.g., Fielding et al., 2012) leading to a number of these conditions being “characterized” by deficits in volitional saccadic eye movements.

The antisaccade task recruits a broad range of cognitive processes, including goal-directed behavior, attention, working memory, learning, and decision-making (Hutton, 2008). Despite the considerable number of studies that have examined the antisaccade task, there is surprisingly little consensus as to which cognitive processes healthy participants use when performing this task. According to classical accounts of the task, antisaccade trials require at least two additional processes compared to prosaccade trials: the suppression of the automatic response to make a visually-guided prosaccade toward the target, and the inversion of the stimulus location into a voluntary motor command to look away from the stimulus (Guitton et al., 1985; Everling and Fischer, 1998). These models argue that this additional processing for antisaccade trials accounts for the increased latency and activity in the oculomotor network for antisaccade vs. prosaccade trials (McDowell et al., 2008). More recent models have moved away from this serial additive factors logic and instead argue for a parallel processing model (Massen, 2004; Munoz and Everling, 2004; Reuter and Kathmann, 2004; Hutton and Ettinger, 2006). These models are conceptually similar to evidence accumulation models proposed earlier (e.g., Ratcliff, 1978; Carpenter, 1981; see Isoda and Hikosaka, 2011). For example, Massen (2004) argues that at stimulus onset, there is competition between the exogenously triggered prosaccade and endogenously triggered antisaccade. If the antisaccade response is computed fast enough and reaches threshold quickly, it wins the competition and the visually-guided saccade is canceled; if it is not computed fast enough, the exogenous prosaccade is executed. Models differ on whether they include an explicit inhibitory process of the prepotent prosaccade response on antisaccade trials. Munoz and Everling (2004) and Isoda and Hikosaka (2011) argue that this inhibitory process is critical to correct antisaccade performance, allowing the antisaccade response to reach threshold before the prosaccade response.

A large and growing body of literature has used functional magnetic resonance imaging (fMRI) and positron emission tomography (PET) to examine the neural correlates of antisaccade performance in both healthy controls and psychiatric/neurologically-impaired populations. The task reliably activates a distributed oculomotor control network, including the frontal eye fields (FEF), supplementary eye field (SEF) and intraparietal sulcus (IPS), among others (see Hutton and Ettinger, 2006; McDowell et al., 2008 for reviews). In general, antisaccade trials activate this oculomotor network to a greater extent than prosaccade trials, and may also recruit additional regions not required on prosaccade trials, such as the anterior cingulate (ACC) and dorsolateral prefrontal cortex (DLPFC; McDowell et al., 2008). Understanding how healthy controls activate the oculomotor network is of great importance, as the task is currently under consideration as a potential endophenotype for schizophrenia (Ettinger et al., 2006; Jablensky, 2009; Ritsner and Gottesman, 2009) and also shows promise as a biomarker for Friedreich Ataxia (Fielding et al., 2010), Huntington's disease (Blekher et al., 2004) and multiple sclerosis (Fielding et al., 2012).

Here, we present a quantitative meta-analysis of existing fMRI and PET studies of the antisaccade and prosaccade tasks. This quantitative meta-analysis is aimed to complement the excellent systematic reviews of the task published recently (Hutton and Ettinger, 2006; Pierrot-Deseilligny et al., 2004; McDowell et al., 2008). The motivation for this quantitative meta-analysis was twofold. Firstly, to examine the consistency of activation for antisaccades and prosaccades across the brain; many studies that examine the antisaccade task in both healthy and impaired individuals tend to focus narrowly on a few *a priori* regions of interest (ROI), such as FEF and IPS, thus ignoring potentially interesting contributors to performance. One obvious example of this is the paucity of studies that include cerebellar ROIs despite evidence that multiple cerebellar regions contribute significantly to antisaccade performance and oculomotor processes in general (Robinson and Fuchs, 2001). Establishing which regions are consistently activated by the task across the brain in healthy individuals will assist in the study and development of the task as a robust endophenotype/biomarker (see also Raemaekers et al., 2007 for a study of test-retest reliability of the antisaccade task). Secondly, fMRI studies that use the ROI approach typically construct their ROIs using anatomy or functional localizer scans. Both approaches are potentially problematic. Anatomical ROIs are problematic as it can be difficult to unambiguously determine the position of functional regions on the basis of anatomy alone. Anatomical localization of a number of regions including FEF and oculomotor IPS in humans differs substantially between individuals (Paus, 1996; Pierrot-Deseilligny et al., 2004; Amiez and Petrides, 2009). With regards to functional ROIs, many studies use a separate localizer scan to establish a study-specific definition of the oculomotor regions. Thus, the definition of the ROI from group maps of the localizer task is necessarily limited by the sample size of the study and of the context sensitivity of the localizer (Friston et al., 2010,^1^). A major goal of the current study was to determine the most consistently activated regions in the antisaccade task in order to create activation masks for restricted ROI analyses in future studies. As such, these ROIs will be created using a much larger sample size than is feasible in many studies, and are also created on the basis of the most consistently activated regions across studies. The resulting FEF ROI can be considered complementary to the previous work of Paus (1996), who reviewed the location of the human FEF on the basis of early PET studies. In this way, the results of this meta-analysis may assist future researchers to overcome the problems associated with using anatomical ROIs and functional localizers.

We hypothesized that cortical regions classically associated with oculomotor control would be consistently activated, including the FEF, SEF and IPS, as well as regions associated with oculomotor control but not widely discussed in fMRI or PET studies, including subcortical (thalamic, striatal), and cerebellar regions. Additionally, we hypothesized that since studies tend to focus their discussions narrowly on regions defined as “oculomotor,” a number of additional regions would also be consistently active, including the visual cortex, and each of these regions would be activated to a greater extent in antisaccade compared to prosaccade trials.

## Materials and methods

### The antisaccade paradigm

In the antisaccade paradigm, participants are required to perform simple saccadic eye movements, either toward a peripheral target when it appears (*prosaccade* trials) or in the mirror opposite direction of the peripheral target (*antisaccade* trials). After the target is extinguished, participants are usually required to make a return saccade back to central fixation (see Hutton and Ettinger, 2006 for a review). Behaviorally, the antisaccade task yields a number of measures: error rate, reaction time/latency of the saccade, the time to correct errors (the time between the erroneous prosaccade to the subsequent corrective antisaccade), amplitude of correct and incorrect saccades and final eye position of the antisaccade. The majority of fMRI studies record video-oculography or infra-red oculography and simply report error rates and latencies.

### Selection procedures

The literature review and selection of manuscripts for the meta-analysis was conducted according to the preferred reporting items for systematic reviews and meta-analyses (PRISMA) guidelines (Moher et al., 2009). These guidelines outline a structured methodology for the performance of a meta-analysis and/or systematic review, with the aim of improving the quality of published reviews.

A systematic search strategy was used to identify relevant studies. First, we carried out a PubMed search to identify putative fMRI studies of the antisaccade task using the search terms “antisaccade” and “fMRI”; only studies published in English were included (this restriction excluded a single manuscript, Fukushima, 2012, published in Japanese). In addition, only studies in humans were selected (excluding four manuscripts). The search was conducted in October 2012, and extended in July 2013 to include PET studies. This search identified 74 manuscripts. In the second step, the reference lists of the articles identified were manually checked for relevant studies not identified in the PubMed literature search; this search identified 14 additional manuscripts. These 84 manuscripts were then screened according to the following criteria: (a) being an original article in a peer-reviewed journal, (b) have used BOLD-fMRI or PET, and (c) have included at least one non-psychiatric/neurologically-impaired group in the age ranges 18–75 years. This selection procedure identified 55 studies.

Five manuscripts were identified as re-analyses of previously published data [Miller et al., 2005 (duplicating Curtis and D'Esposito, 2003); Polli et al., 2008a (duplicating Polli et al., 2005); Roffman et al., 2011a (duplicating Manoach et al., 2007); Velanova et al., 2009 (duplicating Velanova et al., 2008); Hwang et al., 2010 (duplicating Velanova et al., 2008)]. In the case of Miller et al. (2005); Polli et al. (2008a), and Roffman et al. (2011a), the original manuscripts presented data relevant to the current manuscript, so Miller et al. (2005); Polli et al. (2008a) and Roffman et al. were excluded. In the case of Velanova et al. (2008, 2009) and Hwang et al. (2010), only Hwang et al. presented data relevant to the current meta-analysis, so the Velanova et al. (2008, 2009) studies were excluded.

The remaining 51 manuscripts were then screened in more detail and classified as: (a) whole-brain or partial-brain fMRI acquisition, (b) whether, in developmental/psychiatric/neurologically-impaired studies, results for a healthy control group (18–75 years) were shown separately, (c) coordinates in MNI or Talairach space were reported for the contrasts of interest, and (d) voxel-wise or region-of-interest (ROI) analysis. Inclusion of studies into a meta-analysis that use ROI analyses can bias the results (Ragland et al., 2009). Five studies acquired functional scans that did not encompass most of the cortex [Cameron et al., 2009 (11 slices, over the caudate); Connolly et al., 2002, 2005 (6 slices, FEF and parietal lobes only); Medendorp et al., 2005 (9 slices over parietal lobe only); Neggers et al., 2012 (30 slices over FEF and subcortical regions only)]; Brown et al. (2006); Cameron et al. (2012), and Raemaekers et al. (2007) were included because they imaged almost all the cortex and subcortical regions. Six studies did not present analyses for a healthy control group aged 18–75 years separate from a developmental/psychiatric/neurologically impaired group (Raemaekers et al., 2002, 2006; Camchong et al., 2008; Thakkar et al., 2008; Agam et al., 2010; Dyckman et al., 2011), and four studies did not report coordinates for the contrast of interest (Polli et al., 2005; Raemaekers et al., 2005a,b; Lee et al., 2013). Of the remaining 35 manuscripts, eleven studies only reported results from ROI analyses (Muri et al., 1998; Luna et al., 2001; Cornelissen et al., 2002; DeSouza et al., 2002; McDowell et al., 2002; Curtis and D'Esposito, 2003; Raemaekers et al., 2007; Polli et al., 2008b; DeWeijer et al., 2010; Geier et al., 2010; Roffman et al., 2011b), one reported results masked by an orthogonal contrast (Klein et al., 2007) and one reported coordinates from only frontal and parietal cortices (Anderson et al., 2008). In addition, four manuscripts (Dyckman et al., 2007; Curtis and Connolly, 2008; Reuter et al., 2010; Harsay et al., 2011) were excluded as they did not report a simple antisaccade/prosaccade contrast. Note that two studies (Kimmig et al., 2001; Hwang et al., 2010) reported ROI results, however, these ROIs were functionally defined from the results of the reported contrast and so were included in the analysis.

Following this systematic search, 18 studies were included in the meta-analysis (Table 1). Of these studies, ten were block-design, seven were event-related, and one was mixed-design. Sample sizes were modest (range 9–54 subjects), four studies used PET and fourteen used fMRI, three included a developmental/psychiatric/neurologically-impaired group, and two rated subjects on the basis of other factors (smoking, schizotypy) but were otherwise psychiatrically and neurologically healthy. Across all studies the total number of subjects contributing to the meta-analysis is 315.

**Table 1:** **Studies included in the meta-analysis**.

**Study**	**Method**	**Groups**	**Healthy n**	**Age mean**	**Gender M/F**	**Original coordinate space**	**Design**	**Antisaccade vs. baseline**	**Prosaccade vs. baseline**	**Antisaccade vs. prosaccade**	**Notes**
Aichert et al., 2012	fMRI	Healthy	54	26.63 (*SD* 5.50)	36/18	TAL^*^	Blocked	Yes	Yes	Yes	Subjects rated on schizotypy scores
Brown et al., 2006	fMRI	Healthy	10	26 (range 22–33)	4/6	TAL	Event	Yes	Yes	Yes	
Brown et al., 2007	fMRI	Healthy	11	25 (range 20–28)	2/9	TAL	Event	No	No	Yes	
Cameron et al., 2012	fMRI	Parkinson's disease, Healthy	13	64.8 (range 51–74)	7/6	TAL	Event	No	No	Yes	
Chickazoe et al., 2007	fMRI	Healthy	24	Range 20–29	12/12	TAL	Event	No	No	Yes	
Doricchi et al., 1997	PET	Healthy	10	Range 20–26	10/0	TAL	Blocked	Yes	Yes	Yes	
Ettinger et al., 2008	fMRI	Healthy	17	27.82 (range 20–40)	10/7	TAL^*^	Event	Yes	No	No	
Ettinger et al., 2009	fMRI	Healthy	24	Smokers 25.85 (*SD* 6.16) non-smokers 23.36 (*SD* 4.8)	24/0	TAL^*^	Blocked	No	Yes	Yes	Smokers and non-smokers
Ford et al., 2005	fMRI	Healthy	10	28	7/3	TAL	Event	No	No	Yes	
Fukumoto-Motoshita et al., 2009	fMRI	Healthy	18	37.6 (*SD* 4.8)	9/9	MNI	Blocked	Yes	No	No	
Hwang et al., 2010	fMRI	Children, Adolescents, Healthy	27	Range 18–27	11/16	TAL	Mixed	Yes	No	No	
Kimmig et al., 2001	fMRI	Healthy	15	27 (range 20–37)	Not reported	TAL	Blocked	Yes	Yes	No	
Manoach et al., 2007	fMRI	Healthy	21	34.2 (*SD* 12.6)	13/8	TAL^*^	Event	No	No	Yes	
Matsuda et al., 2004	fMRI	Healthy	21	39.2 (SD 10.2)	Not reported	MNI	Blocked	Yes	Yes	Yes	
O'Driscoll et al. (1995)	PET	Healthy	10	26.2 (range 22–39)	4/6	TAL	Blocked	No	No	Yes	
Paus et al. (1993) Expt 2	PET	Healthy	9	Range 19–30	5/4	TAL	Blocked	Yes	Yes	Yes	
Sweeney et al. (1996)	PET	Healthy	11	27 (*SD* 6)	6/5	TAL	Blocked	No	Yes	Yes	The “conditional antisaccade” condition not included here
Tu et al., 2006	fMRI	Schizophrenia, Healthy	10	27.9 (*SD* 3.18)	5/5	MNI	Blocked	Yes	No	No	

* Converted from MNI using Brett transform (http://imaging.mrc-cbu.cam.ac.uk/imaging/MniTalairach). Abbreviations: SD, standard deviation, TAL: Talairach, MNI, Montreal Neurological Institute.

A number of steps were taken to ensure all results included in the meta-analysis were in the same stereotaxic space. The majority of manuscripts reported results in Talairach coordinates (Table 1); those that reported results in MNI space were transformed using the icbm2tal transform (Lancaster et al., 2007). In addition, the four manuscripts that reported results in Talairach space converted from MNI space using the Brett mni2tal transform (http://imaging.mrc-cbu.cam.ac.uk/imaging/MniTalairach) were converted back to MNI space using the inverse tal2mni transform, then converted to Talairach space using the icbm2tal transform.

### Quantitative meta-analysis procedures

Quantitative meta-analysis was conducted using activation likelihood estimation (ALE) as implemented in GingerALE v2.1 (Turkeltaub et al., 2002; Laird et al., 2005a; Eickhoff et al., 2009). ALE is a random effects voxel-based method for finding concordance across manuscripts and does not rely upon author-assigned anatomical labels; rather, it only requires that activation foci are reported in standard stereotaxic space (Laird et al., 2005b; Eickhoff et al., 2009). In ALE, each activation focus from each experiment is modeled as the center of a Gaussian probability distribution, scaled inversely by the square root of the sample size (Eickhoff et al., 2009). That is, the width of the modeled probability distribution reflects the uncertainty of the spatial localization by taking into account between-subject variance. In this way, foci from studies with small *n* will be modeled as a more blurred function with less localizing impact, and foci from studies with large *n* will be modeled as tighter, more highly weighted function (Eickhoff et al., 2009). Following the transformation of the coordinates into probability functions, a whole-brain map is created by assigning a value to every gray-matter voxel in the brain that reflects the probability that a reported foci lies within that voxel (Turkeltaub et al., 2002; Eickhoff et al., 2009). The ALE value tends to be small due to the large number of voxels in the brain (Turkeltaub et al., 2002). The *activation likelihood estimation* map is then thresholded using standard imaging statistical methods (Laird et al., 2005a). Statistical significance was determined using an FDR correction of *p* < 0.05, with minimum cluster size of 200 mm^3^, as implemented in GingerALE v2.1, and described by Laird et al. (2005a) and Eickhoff et al. (2009).

Three separate meta-analyses were conducted: (1) antisaccade greater than fixation or rest, (2) prosaccade greater than fixation or rest, (3) antisaccade greater than prosaccade. Very few studies examined prosaccade greater than antisaccade so this was not considered. Additionally, given that the number of manuscripts reporting (2) was modest (8 studies), a quantitative conjunction or subtraction between (1) and (2) at the meta-analysis level was not possible. For all analyses, coordinates from event-related designs were sourced from analyses locked to the target or response (i.e., not to cues/preparation interval).

## Results

### Antisaccade > fixation and prosaccade > fixation

**Figure 1:**
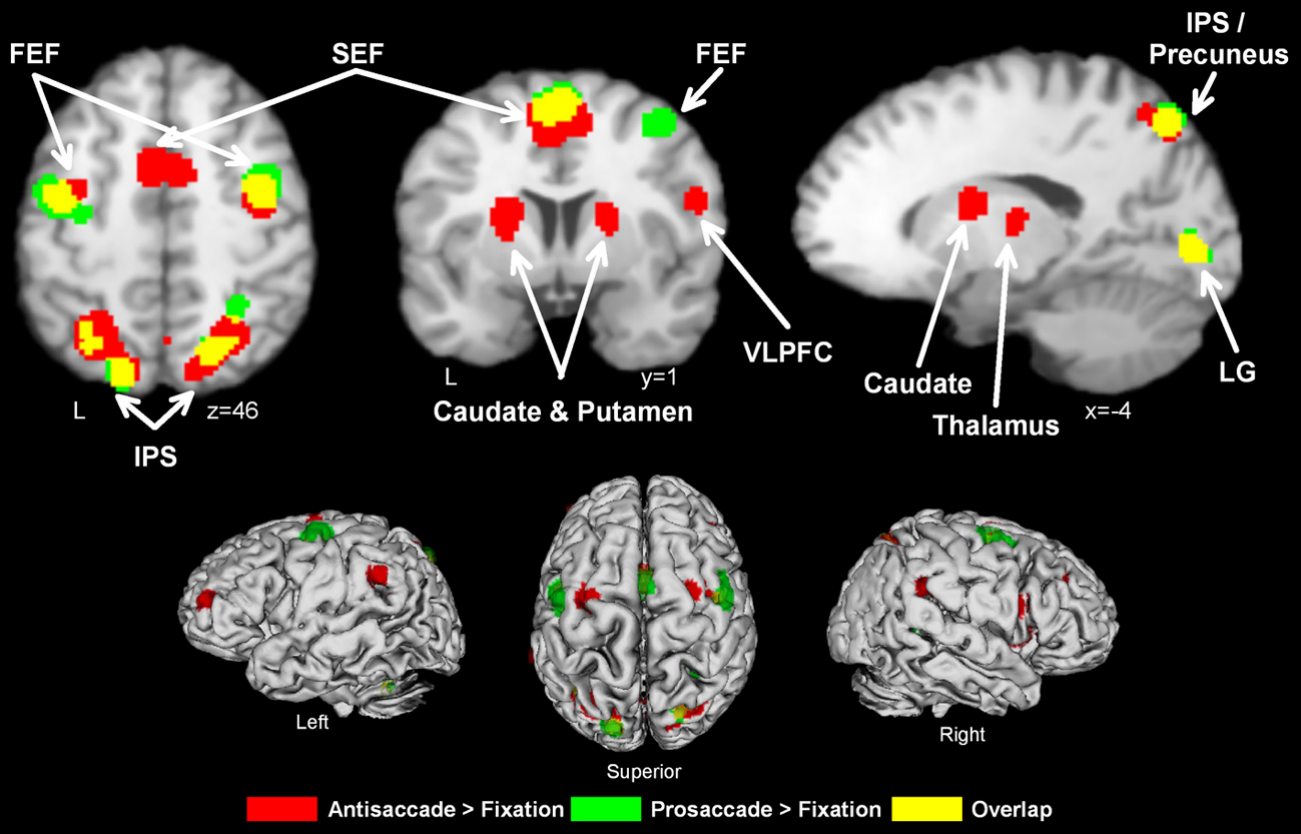
**Activation likelihood maps for antisaccade > fixation (red) and prosaccade > fixation (green)**. Regions where the contrasts overlap are shown in yellow. Abbreviations: FEF, frontal eye field; SEF, supplementary eye field; SPL, superior parietal lobule; IPS, intraparietal sulcus; IFG, inferior frontal gyrus; LG, lingual gyrus.

Ten manuscripts reported foci for antisaccade greater than fixation, yielding 168 total foci. Antisaccade trials consistently activated eighteen regions (Table 2, Figure 1, red). These activations were largely bilateral, and encompassed frontal and SEFs, IPS, thalamus, caudate, putamen, insula, precuneus, and lingual gyrus.

**Table 2:** **Talairach coordinates, volume (mm^3^), ALE values and anatomical labels for the antisaccade > fixation analysis**.

**Cluster #**	**Volume (mm^3^)**	**ALE**	***x***	***y***	***z***	**Macroanatomical label (Functional label, Brodmann area)**
1	3896	0.0321	0	0	52	Medial frontal gyrus (Supplementary eye field, BA 6)
2	3576	0.0280	−24	−62	46	Left intraparietal sulcus (BA 7)
3	2800	0.0234	26	−62	48	Right intraparietal sulcus/precuneus (BA 7)
4	2320	0.0288	10	−18	12	Right thalamus
			18	−4	16	Right caudate
5	1728	0.0313	36	−8	50	Right precentral gyrus/middle frontal gyrus (frontal eye field, BA 6)
6	1712	0.0190	−38	−10	48	Left precentral gyrus/middle frontal gyrus (frontal eye field, BA 6)
7	1248	0.0234	−20	−2	14	Left putamen
8	800	0.0180	58	−38	28	Right supramarginal gyrus (BA 40)
9	752	0.0183	−12	−84	−2	Left lingual gyrus (BA 18)
10	544	0.0185	−12	−18	8	Left thalamus
11	544	0.0201	54	4	20	Right inferior frontal gyrus (ventrolateral prefrontal cortex, BA 9/44)
12	392	0.0155	26	−6	60	Right middle frontal gyrus (frontal eye field, BA 6)
13	320	0.0169	−60	−42	30	Left supramarginal gyrus (BA 40)
14	280	0.0161	−42	44	12	Left middle frontal gyrus (frontal eye field, BA 6)
15	272	0.0147	12	−78	8	Right cuneus (BA 17/18)
16	216	0.0147	48	10	4	Right insula (BA 13)

Brodmann areas are given for the peak of ALE within the cluster.

Eight manuscripts reported foci for prosaccade greater than fixation, yielding 78 foci. Prosaccade trials activated six regions (Table 3, Figure 1, green), including frontal and supplementary eye fields, IPS, and precuneus.

**Table 3:** **Talairach coordinates, volume (mm^3^), ALE values and anatomical labels for the prosaccade > fixation analysis**.

**Cluster #**	**Volume (mm^3^)**	**ALE**	***x***	***y***	***z***	**Macroanatomical label (Functional label, Brodmann area)**
1	2816	0.0326	−2	−4	58	Left medial frontal gyrus (supplementary eye field, BA 6)
2	2104	0.0222	40	−4	50	Right middle frontal gyrus (frontal eye field, BA 6)
3	2008	0.0205	−40	−10	50	Left precentral gyrus/middle frontal gyrus (frontal eye field, BA 6)
4	840	0.0178	18	−68	50	Right intraparietal sulcus (BA 7)
5	800	0.0188	−16	−78	48	Left precuneus/Intraparietal sulcus (BA 7)
6	512	0.0131	−14	−84	−2	Left lingual gyrus (BA 17)
7	440	0.0121	−24	−62	40	Left intraparietal sulcus (BA 40)
8	416	0.0144	28	−52	44	Right intraparietal sulcus (BA 7)

Brodmann areas are given for the peak of ALE within the cluster.

As can be seen from Figure 1 and Table 2, antisaccade > fixation tended to activate a more distributed set of regions than prosaccade > fixation. While there was considerable overlap between the two contrasts, the spatial extent of activation tended to be greater in antisaccade > fixation compared to prosaccade > fixation.

### Antisaccade > prosaccade

**Figure 2:**
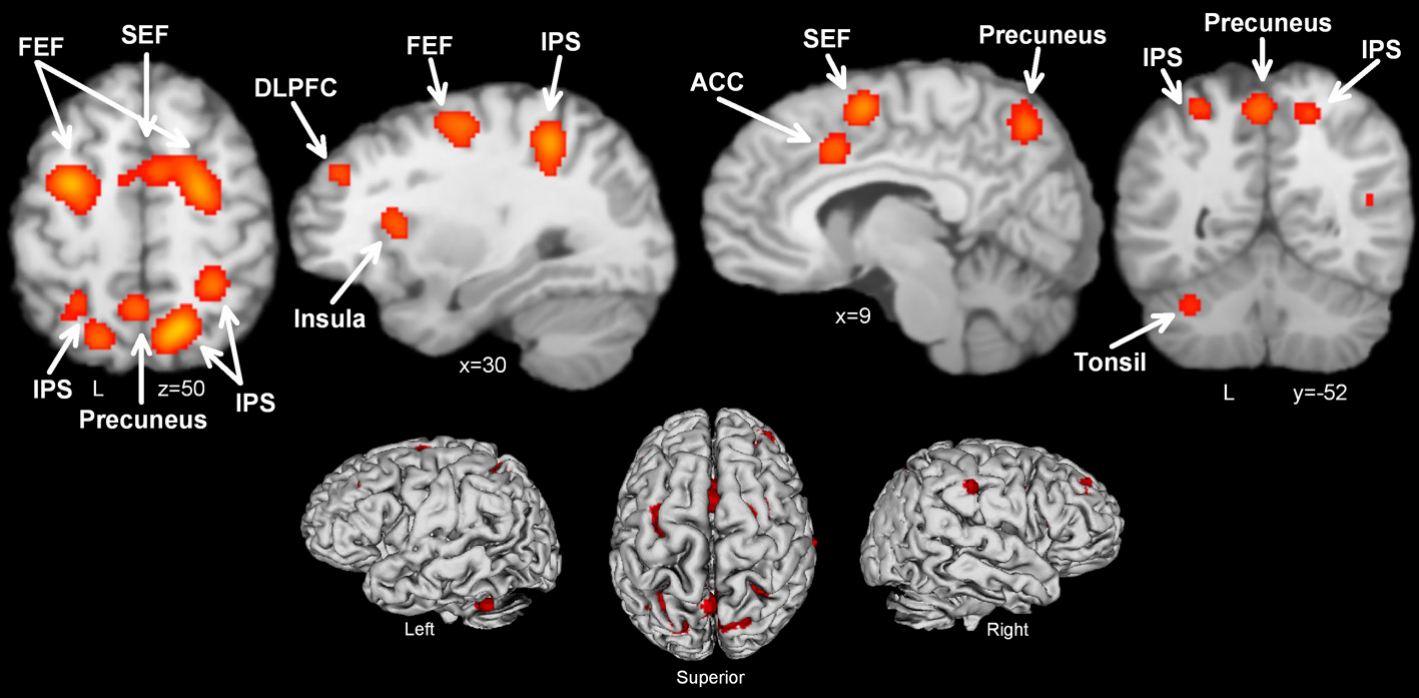
**Activation likelihood maps for antisaccade > prosaccade**. Abbreviations: FEF, frontal eye field; SEF, supplementary eye field; SPL, superior parietal lobule; DLPFC, dorsolateral prefrontal cortex; IPS, intraparietal sulcus; ACC, anterior cingulate cortex.

Thirteen manuscripts reported foci for antisaccade greater than prosaccade, yielding 192 total foci. Antisaccade vs. prosaccade trials (Table 4, Figure 2) consistently activated 13 regions, including frontal and SEFs, IPS, anterior cingulate, DLPFC, precuneus, insula, and cerebellar tonsil. The set of regions obtained in this analysis were broadly compatible but not identical with the regions obtained in the antisaccade > fixation: antisaccade > prosaccade consistently activated subcortical regions whereas antisaccade > fixation did not, also antisaccade > fixation consistently activated anterior cingulate and cerebellum whereas antisaccade > prosaccade did not.

**Table 4:** **Talairach coordinates, volume (mm^3^), ALE values and anatomical labels for the antisaccade > prosaccade analysis**.

**Cluster #**	**Volume (mm^3^)**	**ALE**	***x***	***y***	***z***	**Macroanatomical label (Functional label, Brodmann area)**
1	6528	0.0450	24	−6	54	Right middle frontal gyrus (frontal eye field, BA 6)
			6	0	54	Right medial frontal gyrus (supplementary eye field, BA 6)
2	3600	0.0333	−28	−4	50	Left middle frontal gyrus (frontal eye field, BA 6)
3	3424	0.0369	14	−66	48	Right precuneus (BA 7)
4	2016	0.0285	30	−46	44	Right intraparietal sulcus (BA 7)
5	1536	0.0181	6	10	36	Right anterior cingulate cortex (BA 24)
			−4	16	30	Left anterior cingulate cortex (BA 32)
6	840	0.0198	−2	−56	50	Left intraparietal sulcus (BA 7)
7	832	0.0170	32	42	32	Right superior/middle frontal gyri (dorsolateral prefrontal cortex, BA 9)
8	784	0.0189	−18	−58	50	Left precuneus (BA 7)
9	576	0.0161	30	18	10	Right insula (BA 13)
10	384	0.0125	−26	−54	50	Left intraparietal sulcus (BA 7)
11	386	0.0128	60	−22	32	Right postcentral gyrus (BA 2)
12	344	0.0171	−30	−54	−32	Left cerebellar tonsil
13	304	0.0166	−12	6	40	Left anterior cingulate (BA 32)

## Discussion

The antisaccade task is a classic measure of cognitive and oculomotor control and has been used extensively in the study of executive function deficits in psychiatric and neurologically-impaired groups such as schizophrenia, multiple sclerosis, and Friedrich's ataxia etc. A large body of literature (>50 published manuscripts to date) has used fMRI or PET to study the neural correlates of the task, and has demonstrated that it robustly and reliably activates a distributed cortico-subcortical-cerebellar network. Here, we focused on voxel-wise studies of healthy individuals to establish regions that are consistently activated by the task.

Antisaccade trials vs. fixation consistently activated a broad range of regions classically associated with oculomotor control and often used as a priori ROIs, including bilateral FEF, SEF, IPS, thalamus, caudate, and putamen. Additionally, activation was consistently obtained in precuneus, supramarginal gyrus, inferior frontal gyrus, middle frontal gyrus (in the frontopolar cortex), cuneus, lingual gyrus, and insula. Prosaccade trials vs. fixation also consistently activated the right FEF, SEF, and bilateral IPS. The contrast of most interest to the current study, antisaccade vs. prosaccade trials, revealed that antisaccades indeed consistently activated the oculomotor network of FEF, SEF, IPS, insula to a greater extent than prosaccades, and additionally recruited the ACC, DLPFC, and cerebellar tonsil. These quantitative results confirm the conclusions of recent systematic reviews of the literature (e.g., Hutton and Ettinger, 2006; Sweeney et al., 2007; McDowell et al., 2008).

In the following, we discuss each of the regions activated by antisaccades and their contribution to the oculomotor network for volitional saccades.

### Regions consistently activated in antisaccades and prosaccades

The FEF is positioned at the intersection between the precentral and superior frontal sulci and is cytoarchitectonically defined as a motor cortex (Pierrot-Deseilligny et al., 2004). It is directly involved in oculomotor control via connections with the superior colliculus and the midbrain oculomotor nuclei (Bruce and Goldberg, 1985; Everling and Munoz, 2000). In the antisaccade task, the FEF is thought to be involved in preparing the antisaccade response in advance and the perceptual decision-making processes leading up to the saccade (Ford et al., 2005; Brown et al., 2006, 2007; Ettinger et al., 2008). Pierrot-Deseilligny et al. (2004) argue that it is less involved in visually-guided prosaccades, consistent with our findings that the total volume of FEF activity was larger in antisaccades vs. prosaccades. McDowell et al. (2008) argue that since medial and lateral FEF have different projections to parietal and frontal regions, these regions may differ functionally; they argue that medial FEF may be involved more in volitional antisaccades and lateral FEF may be involved more in visually-guided prosaccades. Our results suggest a refinement of this argument: prosaccade vs. fixation consistently activated lateral FEF (Talairach x = ±40) whereas antisaccade vs. fixation consistently activated both lateral (x = +36/−38) and medial (x = ±26) aspects. This result is reflected in the antisaccade vs. prosaccade contrast as a medial FEF activation only. While further study is required, this result suggests that the lateral FEF may be involved in both volitional antisaccades and visually-guided prosaccades, whereas the medial FEF may be involved more specifically in volitional antisaccades. Future studies should examine this possibility.

The SEF is located on the medial wall in the paracentral sulcus in the human brain (Amiez and Petrides, 2009). It is thought to be the oculomotor extension of the supplementary motor area (Schall, 2002) and typically shows greater activity during cognitively demanding saccades vs. visually-guided saccades (Olson and Gettner, 2002; McDowell et al., 2008). Here, the SEF was consistently activated for both antisaccade and prosaccades, but as hypothesized the extent of the activation was greater for antisaccades, reflected in the consistent activation in the antisaccade vs. prosaccade contrast. In the antisaccade task, the SEF plays a role in the preparation of a voluntary antisaccade as it is maximally activated just before the execution of the saccade (Pierrot-Deseilligny et al., 2004; Ford et al., 2005; Brown et al., 2007). Within competition-based models, it has been argued that the SEF sends the inhibitory signal to suppress the prepotent prosaccade response, giving the antisaccade response time to reach threshold before the prosaccade response (Schlag-Rey et al., 1997; Amador et al., 2004; Boxer et al., 2006). The finding that SEF activity in the preparatory interval predicts accurate performance (Schlag-Rey et al., 1997; Amador et al., 2004) is consistent with this argument.

Antisaccade and prosaccade trials consistently activated the IPS. As hypothesized, antisaccade trials activated the IPS to a greater extent than prosaccade trials. The IPS extends from the postcentral sulcus anteriorly to the parieto-occipital sulcus posteriorly, separating the superior and inferior parietal lobules (Pierrot-Deseilligny et al., 2004; Grefkes and Fink, 2005). The human homolog of the lateral intraparietal area, the parietal eye field (PEF) is located in the posterior part of the IPS and projects to the FEF and superior colliculus (Pierrot-Deseilligny et al., 1991, 2004). IPS activation in the antisaccade task is traditionally linked to the process of vector inversion—that is, the translation of the visual location of the target to the mirror image location (Medendorp et al., 2005; Brown et al., 2006; Nyffeler et al., 2007), thus it is considered not to be critical for the execution of the saccade, rather the ability to accurately perform the saccade to the mirror image location. Note that in this meta-analysis we focused on target or response-locked contrasts, thus demonstrating that the IPS is consistently activated during the saccadic response itself. Most studies argue that this region is not critically involved in preparatory set—i.e., that it is not activated in the pre-target interval (Connolly et al., 2002, 2005; Curtis and D'Esposito, 2003), although this finding is equivocal (DeSouza et al., 2002; Brown et al., 2006). Additionally, Anderson et al. (2008) argue that the IPS also contributes to the inhibition of the prepotent prosaccade response in a *reactive* manner, which contrasts with the proposed *proactive* inhibitory role of the FEF and SEF discussed previously.

### Regions consistently activated in antisaccades but not prosaccades

The ACC was consistently active for antisaccades vs. prosaccades. We found two foci of activity that correspond to the rostral ACC and dorsal ACC (Devinsky et al., 1995; Bush et al., 2000); these foci did not appear to overlap with the cingulate eye field (Gaymard et al., 1998), as the foci were more inferior than reported previously (Amiez and Petrides, 2009). The ACC is known to play a critical role in performance monitoring and conflict detection (MacDonald et al., 2000; Botvinick et al., 2001, 2004; Braver et al., 2001). Polli et al. (2005) conducted a systematic investigation of the role of the rostral and dorsal portions of the ACC in the antisaccade task and reported that during the preparatory phase of the antisaccade trial, correct but not incorrect antisaccade trials showed significant deactivation of the rostral ACC. This was linked to default mode network deactivation, which is considered critical for concentration on the task at hand (Raichle et al., 2001). During the same period, dorsal ACC was active for both correct and incorrect antisaccades, but the magnitude of the activation was related to fewer errors for antisaccade trials. In the later phase of the trial, both rostral and dorsal ACC showed increased activity for incorrect vs. correct antisaccade trials. Polli et al. (2005) concluded that both rostral and dorsal ACC were critical for performance optimization on the task, whereas only rostral ACC was critical for performance evaluation.

Antisaccade vs. prosaccade contrasts consistently activated the DLPFC. Within the oculomotor control network, the DLPFC is considered to take a supervisory role, biasing other oculomotor areas in the service of current behavioral goals (Pierrot-Deseilligny et al., 2003, 2004; Ford et al., 2005; Brown et al., 2007; Ettinger et al., 2008; Hwang et al., 2010). This is consistent with formulations of DLPFC function in other domains including attentional control (Corbetta and Shulman, 2002), task-rule representation (Crone et al., 2006), task-set maintenance and switching (Jamadar et al., 2010a) and working memory (Curtis and D'Esposito, 2003). Here, we found consistent activation for antisaccades > prosaccades in right DLPFC. Lateralization of the antisaccade vs. prosaccade difference in the right hemisphere has been noted previously (Ford et al., 2005; Ettinger et al., 2008) although it has not been rigorously tested, here or elsewhere. Right DLPFC activity in the antisaccade > prosaccade contrast has been linked to a general response inhibition process (Garavan et al., 2006). Likewise, the right VLPFC was consistently active in the antisaccade vs. fixation contrast and has widely been implicated in inhibitory control (Aron and Poldrack, 2006; Jamadar et al., 2010a) and evidence suggests that this region is critical for inhibiting the prepotent response in the oculomotor and somatic motor domains (Chickazoe et al., 2007). Future studies should directly test for lateralization effects in DLPFC during antisaccades.

A number of subcortical regions, including the thalamus, caudate, and putamen were consistently activated in antisaccade vs. fixation. Intriguingly, these regions were not evident in the antisaccade vs. prosaccade contrast even though they were evident in the antisaccade vs. fixation but not prosaccade vs. fixation contrasts. This finding was not attributable to sub-threshold activation likelihood estimates in the antisaccade vs. prosaccade contrast, as reducing the threshold to *p* < 0.10 FDR corrected did not yield significant activation likelihood estimates in these regions. Thus, when using a fixation baseline but not prosaccade baseline the thalamus, putamen, and caudate are consistently activated. The putamen and caudate, collectively known as the *striatum*, are known to play a pivotal role in the cortico-thalamic-basal ganglia networks in both oculomotor (e.g., Isoda and Hikosaka, 2011) and somatic motor (e.g., Parent and Hazrati, 1995) behavior. Briefly, cortical eye fields including the FEF and SEF form three major pathways via the basal ganglia to the superior colliculus: the direct, indirect and hyperdirect pathways (see Isoda and Hikosaka, 2011 for a review). The thalamus is known to play a pivotal role in these networks, and the cortical eye fields receive numerous projections from multiple nuclei in the thalamus, which itself receives multiple inputs from the basal ganglia and cerebellum (Tanaka and Kunimatsu, 2011). Within the thalamus, activation was obtained across multiple thalamic nuclei including the medial dorsal, ventroanterior and ventrolateral nuclei. These form part of the paralaminar portion of the thalamus which is important for oculomotor control (see Tanaka and Kunimatsu, 2011 for a review).

Activation in other regions of the cortico-thalamic-basal ganglia networks known to be important for oculomotor control, including the substantia nigra, globus pallidus, and the superior colliculus itself, was not consistently obtained. These deep small structures can be difficult to image using blood oxygenation level dependent (BOLD) measures, due to the close proximity of these structures to large vasculature (Petit and Beauchamp, 2003; Schneider and Kastner, 2005). Difficulty in imaging deep structures with BOLD fMRI is only exacerbated with the recent move to higher-dimension arrays (>12 channels) in radiofrequency (RF) head coils (Kaza et al., 2011). Despite these challenges, it is possible to optimize the fMRI acquisition and analysis protocols to robustly detect BOLD changes in deep structures like the superior colliculus (see Wall et al., 2009 for a review). Acquisition protocols with in-plane voxel sizes of 1.5 × 1.5 mm at high field strength (> = 3T) have successfully yielded visual and attentional functional activity in the superior colliculus (e.g., Schneider and Kastner, 2009; Krebs et al., 2010). Cardiac gating (Guimaraes et al., 1998) and inclusion of cardiac and respiratory signals as covariates of no-interest in first-level models have also shown success (Wall et al., 2009). Lastly, the fMRI signal of deep structures can be improved by optimizing the temporal properties of the modeled hemodynamic response function (HRF) at the first-level for detection of fMRI changes in deep structures (Wall et al., 2009; Krebs et al., 2010). Given the known importance of deep structures in antisaccade performance and oculomotor control more generally, future studies should explore methods to optimize the acquisition and analysis protocols for detection of signals in these regions.

### Novel findings

The regions discussed so far form the cortico-thalamic-basal ganglia networks that have been identified in systematic investigations of oculomotor control in ROI-based studies of the antisaccade task. In addition to these regions, we also found consistent activation in a number of regions not classically linked to oculomotor control. The regions have been reported in antisaccade studies but are rarely commented upon.

Antisaccade vs. prosaccade trials consistently activated the cerebellar tonsil. This activity is outside of the established oculomotor cerebellar network (Robinson and Fuchs, 2001) and is located in lobule VI of the posterior lobe of the cerebellum. This lobule has been labeled the *cognitive cerebellum*, and it is involved in a range of cognitive processes, including cognitive control tasks. Spatial tasks in particular activate the left lobule VI (Stoodley and Schmahmann, 2010). It is interesting that while the cerebellum is known to be critical for effective oculomotor control as demonstrated by lesion and ataxia studies (Robinson and Fuchs, 2001), these regions are not consistently reported in fMRI or PET studies of the antisaccade task. It is not clear why this is the case. The oculomotor cerebellum may be equally active for antisaccades, prosaccades, and fixation. However, given the known functional role of the cerebellum this seems unlikely. One possibility is that published studies to date have chosen to focus primarily on cortical and subcortical activation, and cerebellar activations remain under-reported. Indeed, despite its known importance in oculomotor control, no study to date has specifically studied fMRI activity of the cerebellum during the antisaccade task. This represents an important direction for future research.

The right insula was consistently activated for antisaccades vs. fixation and antisaccades vs. prosaccades. In addition to its well known role in limbic system and the evaluation of pain, functional neuroimaging studies have shown that the insula is active across multiple cognitive domains, including motor, attention, language, and working memory (Kurth et al., 2010). Recently, it has been argued that the insula interacts with the ACC to identify the most behaviorally salient stimuli, initiating attentional control systems to deal with particularly complex and salient environmental demands (Menon and Uddin, 2010). Given that antisaccade trials require inhibition of probably one of the most prepotent responses in our behavioral repertoire, the finding that the insula is consistently activated for antisaccades is consistent with this model.

Antisaccade vs. fixation consistently activated the precuneus and supramarginal gyrus; precuneus was also consistently activated in antisaccade greater than prosaccade contrasts. These regions lie on the gyral border of the IPS, and given that the morphology of the IPS is known to vary substantially across individuals, one possibility is that the activation in these regions represents the superior medial and inferior lateral extensions of the oculomotor IPS. Nevertheless, the known functions of these regions is compatible with their consistent activation in the antisaccade task: the precuneus has been linked to visuo-spatial information processes compatible with the vector-inversion process postulated in models of antisaccade performance (Selemon and Goldman-Racic, 1988; Cavanna and Trimble, 2006; Dyckman et al., 2007; Reuter et al., 2010) and the inferior parietal lobule, which contains the supramarginal gyrus, is involved in the coding of motor actions for the control of the eyes (Rizzolatti et al., 2006). Lastly, lingual gyrus and cuneus were consistently activated in antisaccade vs. fixation. These regions are involved in visual processing, and it is possible that their increased activity for antisaccades represents increased demand for complex processing. The cuneus has been implicated in visuo-spatial analysis of the environment for arm reaching and eye movements (Shipp et al., 1998; Galletti et al., 1999; Vanni et al., 2001), and the lingual gyrus has been implicated in color processing (Miceli et al., 2001), which many studies use as cues in the antisaccade task. Alternatively, these regions may be consistently activated because of differences in visual stimulation between antisaccade and fixation, or due to movement of the visual environment on the retina with the eye movement in antisaccade trials. As seen in Table 2, these activation foci lay near BA 17 and 18—i.e., near V1 and V2, which is consistent with this interpretation.

### Relationship to models of antisaccade and prosaccade performance

The results of the current meta-analysis provide quantitative support for the spatial locations of the oculomotor regions discussed in previous reviews and models of antisaccades and prosaccades (e.g., Everling and Fischer, 1998; Munoz and Everling, 2004; Hutton and Ettinger, 2006; Sweeney et al., 2007; McDowell et al., 2008; Isoda and Hikosaka, 2011). These models propose that directional and reciprocal connections between these regions underlie correct performance of these tasks. For example, McDowell et al. (2008) argue that visual input enters the brain through the lateral geniculate nucleus of the thalamus, to V1/V2/V3 in visual cortex, and to the SPL and IPS in parietal cortex. Reciprocal connections between parietal cortex and FEF, SEF, DLPFC, and ACC in the frontal cortex underlie the top-down signals that inhibit the reflexive prosaccade, perform vector inversion to determine the location of the planned antisaccade and initiate the antisaccade response. While the results of the current meta-analysis provide support for the consistent activation of these regions during antisaccade and prosaccade trials, given the nature of the analysis we cannot make firm conclusions on the directionality or the interactions between the brain regions identified here. To do so, formal tests of causal models of the task are required; to our knowledge, only a single study has conducted such an analysis. Using Granger causality, Hwang et al. (2010) identified top-down connections from ACC, right DLPFC and VLPFC to cortical and subcortical oculomotor regions during antisaccade but not prosaccade performance. This study represents an important first step to understanding the causal interactions between the neural regions consistently activated in antisaccades and prosaccades.

Surprisingly, we obtained consistent right VLPFC activity in the antisaccade vs. fixation contrast but not antisaccade vs. prosaccade contrast. It is possible that this is related to the fact that most studies that reported the antisaccade vs. prosaccade contrast used an event-related design (6/9 studies). In order to achieve an event-related design, it is necessary to randomize trial types within a single run of trials. Such randomization is known to induce task-switching effects (cf. Rogers and Monsell, 1995), which can change the cognitive effect of interest. For example, switching between tasks that differ in prepotency may induce asymmetrical switch costs (Allport et al., 1994). Interference models of task switching posit that in order to perform the currently relevant task, the currently irrelevant task must be inhibited (Allport et al., 1994; Mayr and Keele, 2000). This task-set inhibition passively dissipates and interferes with subsequent trials. In these models, the increased reaction time and error rate on switch trials occurs because the currently relevant task-set is in a state of inhibition that is carried over from the previous trial. When switching between tasks of unequal difficulty, the prepotent task (here prosaccades) must be strongly inhibited to perform the weaker task (antisaccades). Thus, when switching back to the prepotent task, it is under a high level of inhibition and becomes more difficult to perform. This results in the paradoxical effect that the prepotent task (prosaccade) is more difficult to perform than the weaker task (antisaccade). Asymmetric switch costs have been reported when switching between antisaccades and prosaccades (Mueller et al., 2009).

It is therefore possible that the requirement to switch tasks in event-related but not blocked designs accounts for one of the surprising findings of this study: right VLPFC was consistently active in the antisaccade vs. fixation contrast but not in the antisaccade vs. prosaccade contrast. The right VLPFC is highly implicated in response inhibition (Aron and Poldrack, 2006) and plays a central role in inhibition of the prepotent prosaccade response in cognitive models of the antisaccade task (e.g., Massen, 2004). According to these models, response inhibition is required on antisaccade but not prosaccade trials; therefore these models predict strong right VLPFC activity in the antisaccade vs. prosaccade contrast. In contrast, the asymmetric switch cost phenomenon (Allport et al., 1994) predicts that right VLPFC activity would be larger for the prepotent prosaccade task than the weaker antisaccade task in an event-related design, but larger for antisaccades than prosaccades in a block-design. Two studies included here reported all three contrasts of interest (antisaccade > fixation, prosaccade > fixation, antisaccade > prosaccade) and obtained right VLPFC activity: Aichert et al. (2012; blocked design) and Brown et al. (2006; event-related design). Consistent with models of antisaccade performance (e.g., Massen, 2004), Aichert et al. (2012) obtained right VLPFC activity in both antisaccade vs. fixation and antisaccade vs. prosaccade contrasts but *not* in the prosaccade vs. fixation contrast. This suggests that inhibition of the prosaccade response was required to perform antisaccade trials, but inhibition of the antisaccade response was not required to perform prosaccade trials. In contrast, Brown et al. (2006) obtained right VLPFC activity in the antisaccade vs. fixation contrast but *not* the antisaccade vs. prosaccade contrast. Importantly, right VLPFC was also activated in the prosaccade vs. fixation contrast, consistent with the argument that inhibition of the prosaccade response was required to perform the antisaccade task, and inhibition of the antisaccade response was required to perform the prosaccade task. Thus, when contrasting the two tasks (antisaccade > prosaccade), activation was no longer above threshold in the right VLPFC.

This comparison of results from Aichert et al. (2012) and Brown et al. (2006) is consistent with the argument that right VLPFC activity was not obtained in the meta-analysis of antisaccade vs. prosaccade contrast, due to the requirement to inhibit the currently relevant task in both antisaccade and prosaccade trials in event-related designs. Note that this account does not preclude the possibility of obtaining right VLPFC activity in antisaccade vs. prosaccade contrast, rather it suggests that it may be canceled out if right VLPFC shows similar levels of activation in prosaccades and antisaccades. If right VLPFC is active in both antisaccade and prosaccade trials, but is active to a greater extent in antisaccade trials, some right VLPFC activity may still be obtained, as in Chickazoe et al. (2007). Admittedly, this account is highly speculative, and represents an important direction for future research.

## Conclusions and directions for future research

The motivation for the current meta-analysis was to examine the regions most consistently activated by the antisaccade task. The major cortical areas known to be important for oculomotor control were consistently activated to a greater extent for antisaccades than prosaccades, including the FEF, SEF, IPS, DLPFC, and ACC. Some, but not all of the major subcortical regions known to be implicated in cortico-thalamic-basal ganglia oculomotor networks were consistently activated, including the striatum and thalamus; activation in other regions such as superior colliculus, substantia nigra and globus pallidus was not obtained, possibly due to artifacts and signal loss in deep areas in close proximity to the vasculature. Also, the smoothing required by ALE may dilute the effect sizes in these small structures. In addition to these oculomotor regions, a number of regions not classically associated with oculomotor control, but often involved in tasks of attention, cognitive control, and visuospatial transformations were consistently active in antisaccade trials, including cerebellar tonsil, precuneus, supramarginal gyrus, insula, lingual gyrus, and cuneus. The finding that classic oculomotor cerebellar regions were not consistently activated during any of the contrasts of interest warrants further investigation.

An important direction for future research regards the experimental design of the antisaccade task. Approximately half the studies used a block design whilst the remainder used an event-related design. Block designs are optimal to study sustained activation in response to a task across multiple trials, whereas event-related designs are optimal to study transient activity in response to single trials. Thus, both designs are indexing different components of the task. Additionally, the requirement to randomize trial types to achieve an event-related design can induce task-switching effects (Rogers and Monsell, 1995) and thus asymmetric switch costs (Allport et al., 1994), changing the cognitive effect of interest. Similarly, randomizing tasks of differing difficulty can induce sequence effects that also change the underlying cognitive process of interest (Jamadar et al., 2010b), and manipulating the local probability of an antisaccade or prosaccade task can also affect antisaccade performance (Chiau et al., 2011). Randomizing antisaccade and prosaccade trials affects the magnitude of activation particularly on frontal regions such as the DLPFC and ACC (Dyckman et al., 2007; Manoach et al., 2007) and may affect activity in the right VLPFC (see section Relationship to Models of Antisaccade and Prosaccade Performance). Other manipulations of the basic cognitive paradigm to make it suitable for event-related fMRI can also change the cognitive process of interest (see Ruge et al., 2013 for a discussion of the effects of experimental design on cognitive processing). Given the small number of studies eligible for inclusion in the current meta-analysis, the current study could not examine differences between blocked and event-related designs. With the publication of further studies, future meta-analyses should endeavor to examine such differences.

An important limitation of this study, which is common to all meta-analyses is publication bias (Ferguson and Branick, 2011). Additionally, a large proportion of the studies identified during the systematic search did not report results from voxel-wise analyses, and thus the results reflect consistency of activity across a reduced selection of studies but not for all existing fMRI studies of the task. Note, however, that meta-analysis of even as few as two studies is considered a meaningful analysis, as it can increase the precision of estimates as much as 30% (Cumming, 2012). A possible limitation is that those studies that did report voxel-wise results may have not optimized their scanning protocols to fully cover the cerebellum for all subjects: it is common practice when acquiring the data to align the scan so as to fully cover cortical (premotor) areas which sometimes exclude the most ventral parts the cerebellum in subjects whose brains are larger than the FOV. Lastly, our use of an FDR correction is considered conservative compared to other thresholding procedures (e.g., cluster-level correction; Chumbley et al., 2010; Eickhoff et al., 2012). We feel that this is acceptable as Chumbley et al., and Eickhoff et al.'s results suggest this is likely to have resulted in smaller activation clusters compared to cluster-level thresholds, rather than yielding false positives or negatives. Despite these limitations, this study establishes that the antisaccade task robustly and consistently activates a broad range of oculomotor regions, making it particularly suitable for the study of endophenotypes and biomarkers for psychiatric and neurological disorders (e.g., Jablensky, 2009; Ritsner and Gottesman, 2009; Fielding et al., 2010, 2012). In addition, the meta-analytic activation maps we report here will be of value for decision neuroscience research, where oculomotor processes are increasingly of interest (e.g., Schall, 2001).

In conclusion, the current meta-analysis established the most consistent activation in fMRI studies of the antisaccade and prosaccade tasks, revealing a number of regions that are consistently active to a greater extent for antisaccade than prosaccade trials. While the number of studies eligible to be included in this meta-analysis was small and the results should thus be considered preliminary, the results of this systematic review reveal that antisaccade and prosaccade trials consistently activate a distributed network of regions both within and outside the classic definition of the oculomotor network. We suggest that future studies should acquire whole-brain functional images and report voxel-wise results to make the literature more amenable to meta-analysis. In this way, future systematic reviews can conduct meta-analytic contrasts and/or meta-regressions across studies. Future studies relying on ROIs to study antisaccade-related fMRI can use the regions identified here to construct functional ROIs and eliminate the need for functional localizer scans whilst also allowing ROI definitions to be based on larger samples than routinely recruited in functional studies. Interested researchers can download masks of the regions identified here for use as ROI definitions from http://dx.doi.org/10.5072/03/51230AD72FDF9.

### Conflict of interest statement

The authors declare that the research was conducted in the absence of any commercial or financial relationships that could be construed as a potential conflict of interest.

## Footnotes

^1^For an interesting exchange on the use of functional localizers to define ROIs, see Friston et al. (2010), Saxe et al. (2010), and Friston and Henson (2010).
